# PDGF‐D Promotes Epithelial–Mesenchymal Transition of Glioma Cells Through the NF‐κB/NOTCH1 Pathway

**DOI:** 10.1002/cam4.71002

**Published:** 2025-06-18

**Authors:** Yao Li, Yao Zhao, Minghao Shi, Xiaoshan Ma, Mingbo Jia, Zhongjun Shen, Xiaoyi Liu, Yunqian Li, Liyan Zhao

**Affiliations:** ^1^ Department of Blood Transfusion Second Hospital of Jilin University Changchun China; ^2^ Department of Neurosurgery First Hospital of Jilin University Changchun China

**Keywords:** EMT, glioma, NF‐κB, NOTCH1, PDGF‐D

## Abstract

**Background:**

Platelet‐derived growth factor‐D (PDGF‐D) is expressed at high levels in various tumors and is involved in epithelial–mesenchymal transition (EMT) and the malignant behavior of cancer cells. However, its role in glioma progression and the underlying molecular mechanisms remain unclear.

**Methods:**

We used data from the Chinese Glioma Genome Atlas to evaluate the correlation among PDGF‐D expression, tumor grade, and phenotype of glioma. The in situ expression of PDGF‐D in clinical glioma specimens was analyzed through immunohistochemistry. Colony formation assays and transwell assays were performed for functional evaluation of glioma cell lines with PDGF‐D knockdown or overexpression. Western blotting and RT‐qPCR were conducted to explore molecular mechanisms.

**Results:**

PDGF‐D was significantly upregulated in high‐grade glioma and was associated with the malignant phenotype and poor prognosis. Knocking down PDGF‐D in the LN18 glioma cell line reduced the expression of phosphorylated p65 and NOTCH1 and inhibited clonal proliferation, migration, invasion, and the EMT program. In contrast, inhibiting p65 phosphorylation in glioma cells overexpressing PDGF‐D led to the downregulation of NOTCH1 and reversed EMT.

**Conclusion:**

PDGF‐D promotes the invasion and migration of glioma cells by activating the NF‐κB/NOTCH1 pathway.

## 1 Introduction

Gliomas are highly malignant and invasive tumors of the brain and the most prevalent primary intracranial malignancy [1, 2]. Despite advances in chemo/radiotherapy and surgical resection techniques, the survival rates of glioma patients remain low. Given the invasive nature of gliomas, some neoplastic cells can remain in the surrounding tissues even when the resected area extends beyond the area of visible tumor involvement. These residual tumor cells are responsible for recurrence, which is one of the main reasons for the treatment recalcitrance of gliomas [3, 4]. The metastatic potential of tumor cells depends on epithelial–mesenchymal transition (EMT), a biological process wherein epithelial cells lose polarity and intercellular adhesion, and gain the migratory and invasive characteristics of mesenchymal cells [5, 6, 7, 8, 9, 10]. EMT is triggered by platelet‐derived growth factor (PDGF)‐D and transforming growth factor‐β [11, 12], which activate the downstream Snail, Slug, ZEB1/2, and Twist1/2 transcription factors, resulting in the upregulation of mesenchymal markers (N‐cadherin, vimentin, and β‐catenin) and the concomitant downregulation of epithelial markers (E‐cadherin) [6, 13, 14]. Li et al. found that inhibiting TGF‐β can induce mesenchymal‐to‐epithelial transformation (MET) in glioblastoma cells and restore the turnover of TGF‐β‐triggered tight junction proteins, thereby decreasing the mobility of glioblastoma cells. Furthermore, TGF‐β also redistributes intermediate filaments and vimentin, which can impact mesenchymal type cells [15]. PDGF‐D harbors a PDGF/vascular endothelial growth factor homologous domain, which is common to other PDGF members and plays a crucial role in its spatial conformation, along with the N‐terminal CUB domain. When activated, PDGF‐D is cleaved in the hinge region between these two domains and separated from the CUB domain, following which two growth factor domains form an active PDGF‐DD homodimer. The latter then binds to its receptor PDGFR‐ββ and activates downstream signaling pathways, eventually promoting cellular proliferation and invasion [16, 17, 18]. PDGF‐D is expressed in various tumor cells and regulates EMT [19, 20]. Following upregulation of PDGF‐D, tumor cells attain an elongated or irregular fibroblast shape along with numerous pseudopodia, lose polarity, and show increased migration and invasion capacity. These phenotypic changes are driven by the upregulation of EMT‐related transcription factors and mesenchymal markers and a concomitant downregulation in epithelial markers [11, 14, 21]. Furthermore, overexpression of PDGF‐D in glioma cells has been associated with malignant progression, although the exact role of PDGF‐D in EMT remains unknown [22, 23].

The NOTCH1 signaling pathway mediates cellular processes including proliferation, differentiation, apoptosis, invasion, and migration of tumor cells [24, 25, 26]. Upon binding to its ligands, NOTCH1 is cleaved and releases the Notch intracellular domain (NICD), which forms a complex with the DNA‐binding protein RBP‐J‐κ and transcriptionally activates target genes that regulate key biological events [27]. NOTCH1 is upregulated in human glioma cells and its high expression levels correlate with tumor occurrence and development [28, 29]. In addition, the EMT process of tumor cells is frequently accompanied by the activation of the NOTCH1 pathway. NOTCH1 inhibition in breast cancer cells suppressed their migration and invasion capacity via upregulation of E‐cadherin and downregulation of N‐cadherin and Snail. Likewise, activation of the NOTCH1/TWIST1 pathway in colon cancer cells can induce EMT [11, 30]. However, the involvement of NOTCH1 in the EMT of glioma cells and the underlying mechanisms are unclear.

PDGF‐D is known to activate the NOTCH1 pathway, although the exact mechanism is unknown [11]. Zhou et al. confirmed that NF‐κB can transcriptionally activate NOTCH1 upon binding to its promoter region and increase NOTCH1 expression [31]. Furthermore, Romashkova et al. found that the PDGF‐D PDGFR‐β interaction can induce IκB kinase (IKK), which in turn activates NF‐κB and promotes the nuclear translocation and transcriptional activity of p‐p65 [32]. Accordingly, we hypothesized that PDGF‐D can upregulate NOTCH1 in glioma cells by activating NF‐κB p65, resulting in the EMT and metastasis of glioma cells.

## 2 Materials and Methods

### 2.1 Patients and Tissue Samples

Tumor specimens were collected from 70 glioma patients during surgical resection at the Department of Neurosurgery of the First Hospital of Jilin University. The patients had not undergone chemotherapy or radiotherapy prior to surgery, and the diagnosis of glioma was confirmed by postoperative histopathological examination. Of the 70 patients, 35 had low‐grade glioma (WHO I‐II) and 35 had high‐grade glioma (WHO III‐IV), and each group included 18 female and 17 male patients. This study was approved by the Ethics Committee of the First Hospital of Jilin University, and all patients provided written informed consent.

### 2.2 Immunohistochemistry (IHC)

The paraffin tissue sections were cleared in xylene, hydrated, heated in citrate buffer to unmask antigens, and then incubated with hydrogen peroxide to block endogenous peroxidases. Following overnight incubation with primary antibodies at 4°C, the sections were probed with HRP‐conjugated sheep anti‐mouse/rabbit IgG for 30 min at room temperature, incubated with DAB to develop color, and counterstained with hematoxylin. The tissue sections were then sealed and observed under a microscope. Five random fields were analyzed in each sample using Image J software.

### 2.3 Cell Culture

The human glioma cell lines LN229, U118, U87, and T98 were purchased from iCell Bioscience Inc. (Shanghai, China), and the 293 T cells and human glioma LN18 cells were provided by the Neurosurgery Laboratory of the First Hospital of Jilin University. The LN18, LN229, U118, and 293 T cells were maintained in Dulbecco's modified Eagle medium containing 5% fetal bovine serum (FBS) and 1% penicillin/streptomycin (PS), and complete MEM (10% FBS, 1% PS, and 1% sodium pyruvate) was used to culture the U87 and T98 cells. The cell cultures were maintained at 37°C in a 5% CO_2_ incubator. In addition, all cell lines were authenticated by short‐tandem repeat analysis and routinely tested for mycoplasma.

### 2.4 Reagents and Antibodies

Delta like 4 (Dll4) was purchased from Peprotech (#140–07‐25ug) and diluted to 0.1 μg/mL with culture medium. The NOTCH pathway inhibitor DAPT was purchased from MCE (#HY‐13027) and diluted with culture medium to 12.5 μM. BAY 11–7082 was purchased from Absin (#O09D07) and diluted to 5 μM with culture medium. Anti‐PDGF‐D antibody was purchased from Abcam. The antibodies for PDGFR‐β (#3169), MMP‐2 (#40994), N‐cadherin (#13116), E‐cadherin (#3195), Slug (#9585), β‐catenin (#8480), vimentin (#5741), NICD (#3608), and Hairy and Enhancement of Split 1 (Hes1) (#11988) were purchased from CST and were diluted 1:1000 for western blotting and 1:200 for IHC. The anti‐GAPDH antibody was purchased from OriGene (#TA802519) and diluted 1:5000 for western blotting. Anti‐p‐p65 antibody was purchased from Affinity (#AF2006) and diluted 1:1000 for western blotting and 1:200 for IHC. Anti‐p‐IκB antibody was purchased from Abmart (#PC2513) and diluted 1:1000 for western blotting. Goat anti‐rabbit and anti‐mouse IgG were purchased from Yeasen, and both were diluted 1:5000 for western blotting.

### 2.5 Western Blotting

The suitably treated cells were sonicated in RIPA lysis buffer with a cocktail of protease and phosphatase inhibitors and then left on ice for 30 min. The lysates were cleared by centrifuging at 14000 rpm for 30 min, and the supernatants were collected. The protein content was then measured using a BCA kit, and equal amounts of protein per sample were denatured in 5× SDS protein loading buffer at 95°C for 5 min. The protein samples were electrophoresed on SDS‐PA gels, and the bands were transferred to PVDF membranes. After blocking with skim milk, the blots were incubated overnight with the appropriate primary antibodies at 4°C and subsequently with the secondary antibody for 1 h at room temperature. The positive bands were visualized using an enhanced chemiluminescence agent, and their grayscale values were calculated using ImageJ software.

### 2.6 RT‐qPCR

Total RNA was extracted using Trizol (Biosharp) and reverse‐transcribed with Hifair III 1st Strand cDNA Synthesis SuperMix as per the kit instructions. RT‐PCR was performed using the Hieff PCR SYBR Green Master Mix, and the 2^‐ΔΔCt^ method was used to calculate relative gene expression levels. The primer sequences are listed in Table 1.

**TABLE 1:** Primer sequences used in RT‐qPCR.

Primer	Sequences (5′‐3′)
PDGF‐D	F: GTGGAGGAAATTGTGGCTGT
	R: CGTTCATGGTGATCCAACTG
NOTCH1	F: CACTGCGAGGTCAACACAGA
	R: GTCCACATCGTACTGGCACA
GAPDH	F: TCGGAGTCAACGGATTTGGT
	R: TTCCCGTTCTCAGCCTTGAC

Abbreviations: F, forward; R, reverse.

### 2.7 Cell Transfection

The PDGF‐D shRNA and PDGF‐D cDNA plasmids were designed and synthesized by Transfer Bio. The plasmids were constructed using the pTSBX‐CMV puro vector, and a FLAG tag sequence was added after the CDS region of PDGF‐D in the PDGF‐D overexpression plasmid (NM‐025208). The target sequences are as follows: PDGF‐D shRNA1, forward 5‐AUCCGAAACCAGUACCAUU‐3, reverse 5‐AAUGGUACUGGUUUCGGAU‐3; PDGF‐D shRNA2, forward 5‐CAGGCGAGAUGAGAGCAAU‐3, reverse 5‐AUUGCUCUCAUCUCGCCUG‐3 NC, forward 5‐UUCUCCGAACGUGUCACGU‐3, reverse 5‐ACGUGACACGUUCGGAGAA‐3. The lentiviruses were packaged in 293 T cells. The LN18 and U87 cells were respectively infected with the sh‐PDGF‐D and OE‐PDGF‐D lentiviruses.

### 2.8 Colony Formation Assay

The suitably treated cells were harvested and seeded in six‐well plates in complete medium at the density of 1000 cells/well. The plates were gently shaken to evenly distribute the cells, and then incubated at 37°C in a 5% CO_2_ incubator for 10 days. Once colony growth was visible, the culture medium was discarded, and the clonal masses were fixed with 4% paraformaldehyde for 30 min and stained with 0.1% crystal violet dye for 10 min. The colonies were photographed and counted at low magnification under a microscope.

### 2.9 Transwell Assay

The transfected cells were seeded into the upper chambers of transwell inserts in serum‐free medium at the density of 5 × 10^4^ cells/200 μL/well, and the lower chambers were filled with 700 μL complete medium. The medium was discarded after 48 h, and the cells that migrated to the bottom surface of the membrane were fixed with 4% paraformaldehyde for 30 min and stained with 0.1% crystal violet for 10 min. The stained cells were photographed and counted to determine migration capacity. For the invasion assay, the upper chambers were precoated with 200 μg/mL Matrigel in serum‐free medium, and the above steps were repeated.

### 2.10 In Vivo Experiments

Four‐to‐six weeks old BALB/c‐nu mice were kept in specific pathogen‐free conditions. U87‐OE‐VEC and U87‐OE‐PDGF‐D cells were mixed with Matrigel in a 2:1 ratio, and approximately 4 × 10^6^ cells were injected subcutaneously into each mouse. Tumors were detected within a week after injection. The mice inoculated with the U87‐OE‐PDGF‐D cells were randomly divided into the control and treatment groups and injected intraperitoneally with 2.5 mg/mL DAPT (100 mg/kg) and an equal volume of physiological saline respectively on alternate days. The mice were euthanized following eight injections, and their tumors were removed. The tumor dimensions were measured using calipers, and the volume was calculated using the following formula: *V* (cm^3^) = length (cm) × width^2^ (cm^2^) × 0.5. The tumor tissues were then fixed overnight with paraformaldehyde, embedded in paraffin, and cut into sections for immunostaining.

### 2.11 Statistical Analysis

All statistical analyses were performed using SPSS 20.0 and the graphs were plotted using GraphPad Prism 8.0. Unpaired *t*‐test was used to compare two groups of samples, and analysis of variance and SNK‐q tests were performed for pairwise comparisons within multiple groups. The Spearman method was used for correlation analysis. Unless otherwise specified, all experiments were repeated three times. *p* < 0.05 was considered statistically significant.

## 3 Results

### 3.1 PDGF‐D is Highly Expressed in Glioma Cells and Promotes Proliferation

**FIGURE 1:**
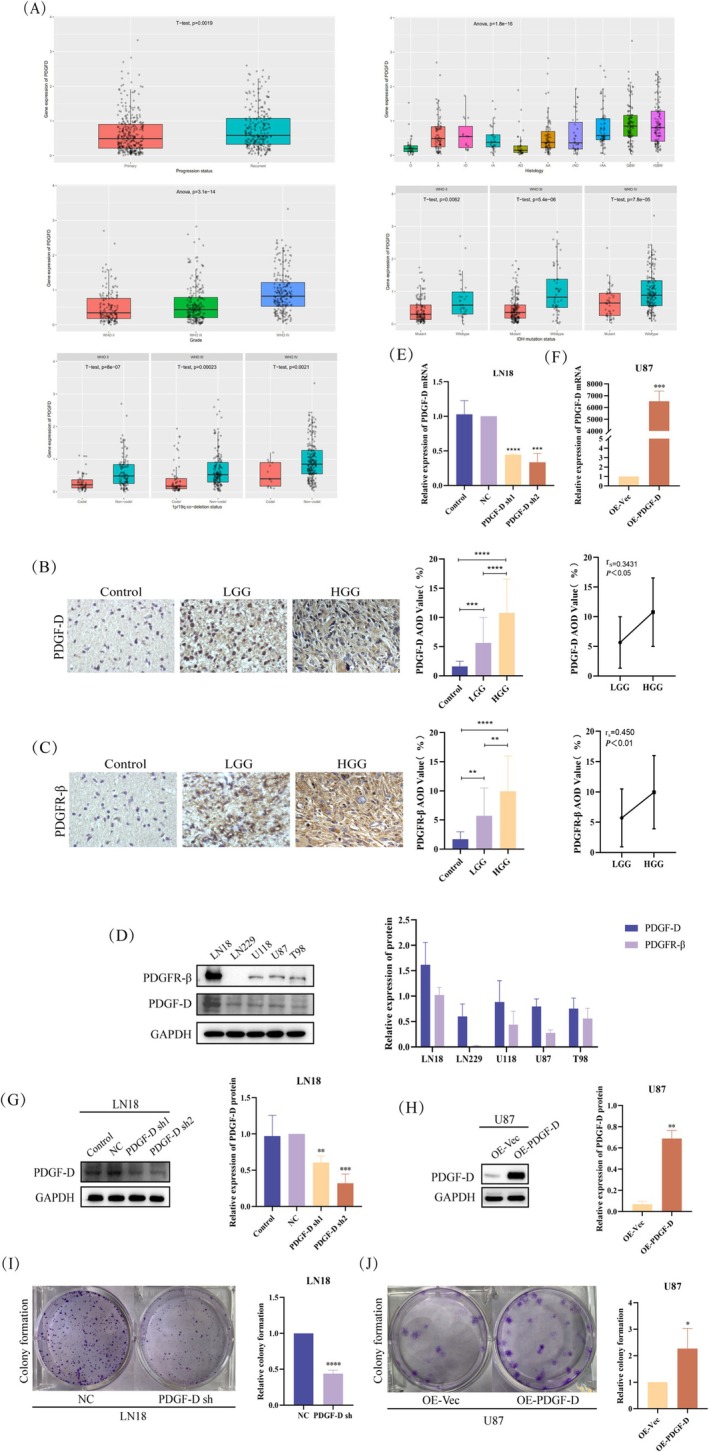
PDGF‐D was highly expressed in glioma and promoted proliferation. (A) CGGA data showing the correlation between PDGF‐D expression in glioma and the degree of malignancy and prognosis. (B, C) Representative immunostaining images showing in situ expression of PDGF‐D and PDGFR‐β in glioma tissues and normal brain tissues. The brown‐stained regions correspond to positive staining. Images are 400× magnified. (D) PDGF‐D and PDGFR‐β protein expression in the indicated glioma cell lines. (E, G) Changes in PDGF‐D mRNA and protein expression after stable transfection of PDGF‐D sh1 and PDGF‐D sh2 in LN18 cells. (F, H) Changes in PDGF‐D mRNA and protein expression levels in U87 cells overexpressing PDGF‐D. (I) Colonies formed by the control and PDGF‐D‐knockdown LN18 cells. (J) Colonies formed by the control and PDGF‐D‐overexpressing U87 cells. *p < 0.05, **p < 0.01, ***p < 0.001, ****p < 0.0001.

The data from the Chinese Glioma Genome Atlas (CGGA) indicated high expression levels of PDGF‐D in various types of gliomas. Furthermore, PDGF‐D expression was positively correlated with the WHO grade. For instance, the recurrent gliomas expressed higher levels of PDGF‐D compared to primary gliomas. Moreover, PDGF‐D expression was significantly higher in gliomas expressing wild‐type isocitrate dehydrogenase (IDH) compared to the IDH‐mutant gliomas, and in 1p‐19q non‐codeletion gliomas versus 1p‐19q co‐deletion gliomas (Figure 1A).

Immunostaining of the clinical specimens indicated higher expression of PDGF‐D in the glioma tissues compared to normal brain tissues (*p* < 0.0001). In addition, the high‐grade glioma tissues showed more intense PDGF‐D expression compared to the low‐grade counterparts (*p* < 0.0001). Spearman analysis further indicated a positive correlation between PDGF‐D expression and the WHO grade of gliomas (*p* < 0.05) (Figure 1B). Likewise, PDGFR‐β was upregulated in the glioma tissues (*p* < 0.0001), especially in the high‐grade gliomas compared to the low‐grade gliomas (*p* < 0.01), and showed a positive correlation to the WHO grade (*p* < 0.01) (Figure 1C). In line with the above, both PDGF‐D and PDGFR‐β showed varying expression levels in different glioblastoma cell lines (Figure 1D). To verify the role of PDGF‐D in glioma progression, we respectively silenced and overexpressed the gene in LN18 cells and U87 cells. PDGF‐D knockdown (PDGF‐D^KD^) and overexpression (PDGF‐D^OE^) in the respective cell lines were assessed by RT‐qPCR and western blotting (Figure 1E–H). As shown in Figure 1I, knocking down PDGF‐D in LN18 cells significantly reduced the number and size of colonies (*p* < 0.0001) (Figure 1I). However, PDGF‐D overexpression in U87 cells resulted in larger and more numerous colonies (*p* < 0.05) (Figure 1J).

### 3.2 PDGF‐D Promotes the EMT of Glioma Cells by Upregulating NOTCH1

**FIGURE 2:**
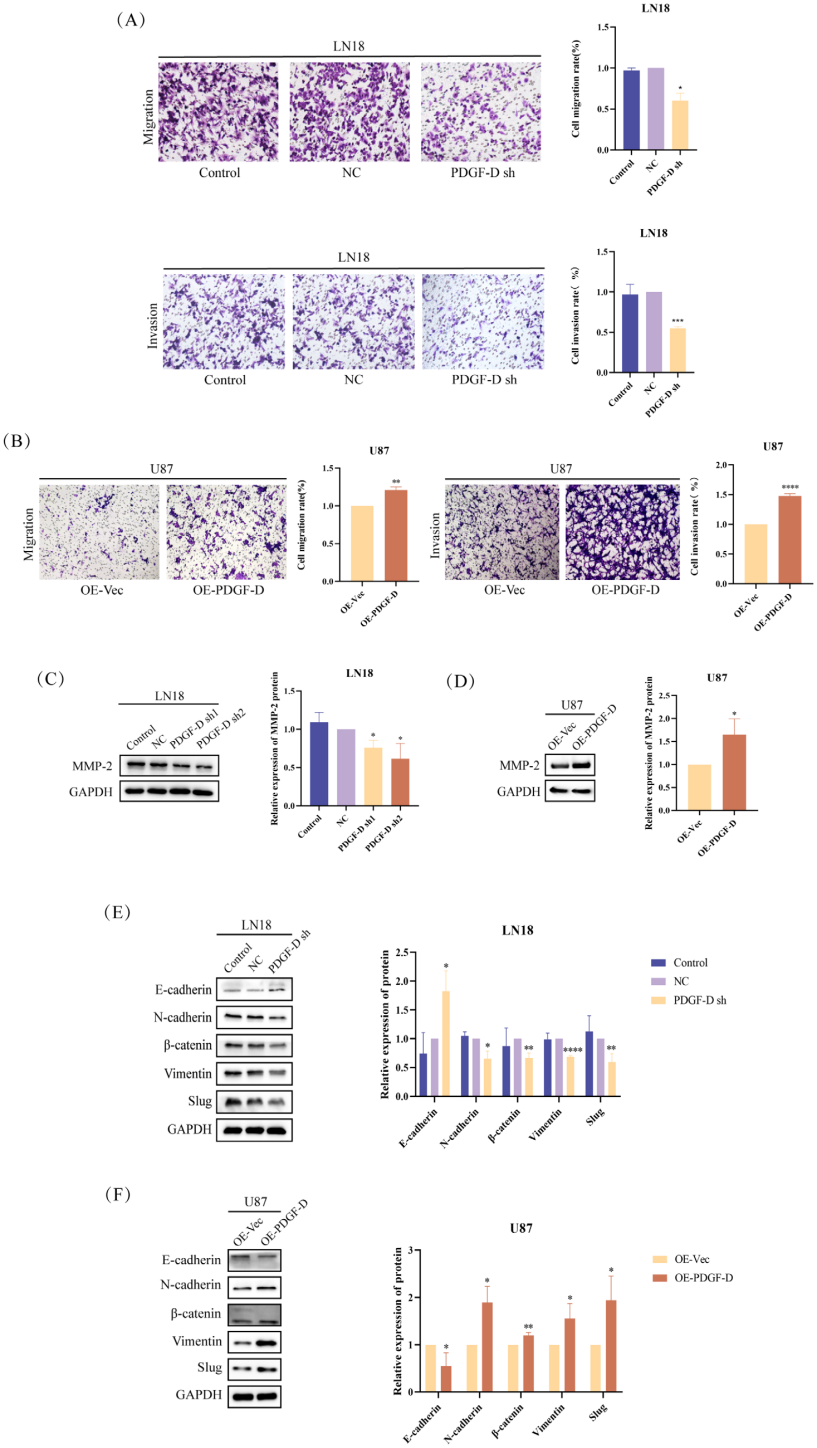
PDGF‐D promoted the EMT of glioma cells. (A) The effect of PDGF‐D knockdown on the migration and invasion of LN18 cells. (B) The effect of PDGF‐D overexpression on the migration and invasion of U87 cells. (C, E) The effect of PDGF‐D knockdown on the expression of MMP‐2 and EMT‐related proteins in LN18 cells. (D, F) The effect of PDGF‐D upregulation on the expression of MMP‐2 and EMT‐related proteins in U87 cells. *p < 0.05, **p < 0.01, ****p < 0.0001.

PDGF‐D knockdown diminished the migration capacity of LN18 cells compared to that of control (NC) cells in the transwell assay (*p* < 0.05) (Figure 2A). Conversely, overexpression of PDGF‐D in U87 cells increased the number of cells passing through the transwell chambers (*p* < 0.001) (Figure 2B). To gain further insights into the potential molecular mechanisms, we analyzed the expression levels of EMT‐related proteins. The MMP‐2 protein was downregulated in the PDGF‐D^KD^ LN18 cells compared to the NC group (*p* < 0.05) (Figure 2C). In addition, the EMT‐related transcription factor slug (*p* < 0.01) and mesenchymal markers such as vimentin (*p* < 0.0001), β‐catenin (*p* < 0.01), and N‐cadherin (*p* < 0.05) were also downregulated, whereas the epithelial marker E‐cadherin was upregulated in the PDGF‐D^KD^ cells (*p* < 0.05) (Figure 2E). In contrast, PDGF‐D overexpression in the U87 cells activated the EMT program, as indicated by an increase in MMP‐2 (*p* < 0.05) (Figure 2D), slug (*p* < 0.05), β‐catenin (*p* < 0.01), and N‐cadherin (*p* < 0.05) and a decrease in E‐cadherin (*p* < 0.05) (Figure 2F).

**FIGURE 3:**
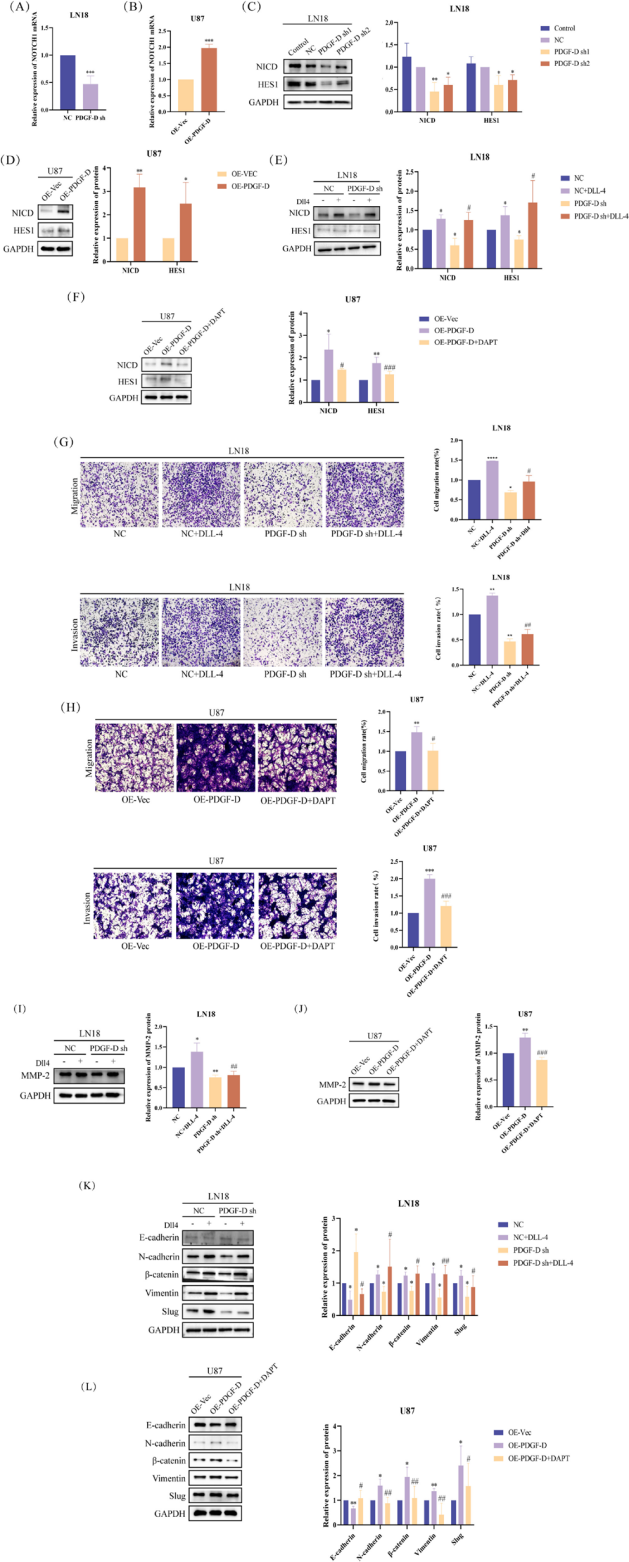
PDGF‐D promoted EMT in glioma cells via NOTCH1. (A, C) Effect of PDGF‐D knockdown on NOTCH1 mRNA and NICD protein expression in LN18 cells. (B, D) Effect of PDGF‐D overexpression on NOTCH1 mRNA and NICD protein expression in U87 cells. (E) Effect of Dll4 on NICD protein expression in LN18 cells with PDGF‐D knockdown. (F) Effect of DAPT on NICD protein expression in U87 cells overexpressing PDGF‐D. (G) Effect of Dll4 on the migration and invasion of LN18 cells with PDGF‐D knockdown. (H) Effect of DAPT on migration and invasion of U87 cells overexpressing PDGF‐D. (I, K) Effect of Dll4 on the expression levels of MMP‐2 and EMT‐related proteins in LN18 cells with PDGF‐D knockdown. (J, L) Effect of DAPT on the expression levels of MMP‐2 and EMT‐related proteins in U87 cells overexpressing PDGF‐D. *p < 0.05, **p < 0.01, ***p < 0.001, ****p < 0.0001 vs. NC or OE‐Vec. ^#^ *p* < 0.05, ^##^ *p* < 0.01, ^###^ *p* < 0.001 vs. PDGF‐D sh or OE‐PDGF‐D.

PDGF‐D knockdown also downregulated NOTCH1 mRNA (*p* < 0.001) and NICD protein (*p* < 0.05), along with the HES1 protein (*p* < 0.05) (Figure 3A,C), a downstream target of NOTCH1. As expected, overexpression of PDGF‐D had the opposite effects (*p* < 0.05) (Figure 3B,D). To clarify whether NOTCH1 is involved in PDGF‐D‐mediated EMT in glioma cells, we activated NOTCH1 in the PDGF‐D^KD^ LN18 cells with Dll4 and inhibited NOTCH1 cleavage in the PDGF‐D^OE^ U87 cells using DAPT. As shown in Figure 3E, Dll4 increased the levels of NICD and HES1 in PDGF‐D^KD^ LN18 cells compared with the untreated cells (*p* < 0.05). However, addition of DAPT downregulated NICD and HES1 in the PDGF‐D^OE^ U87 cells (*p* < 0.05) (Figure 3F). Furthermore, NOTCH1 activation reversed the inhibitory effect of PDGF‐D knockdown on the migration (*p* < 0.05) and invasion (*p* < 0.01) of LN18 cells (Figure 3G), while NOTCH1 inhibition neutralized the stimulatory effects of PDGF‐D overexpression (*p* < 0.05) (Figure 3H). In line with the above findings, Dll4 restored the expression of MMP‐2 (*p* < 0.01) (Figure 3I), slug (*p* < 0.05), vimentin (*p* < 0.01), β‐catenin (*p* < 0.05), and N‐cadherin (*p* < 0.05) and downregulated E‐cadherin (*p* < 0.05) (Figure 3K) in the PDGF‐D^KD^ LN18 cells, while DAPT treatment inhibited the EMT program in U87 cells overexpressing PDGF‐D (*p* < 0.05) (Figure 3J,L). Taken together, PDGF‐D promotes the EMT, migration, and invasion of glioma cells by activating NOTCH1 signaling.

### 3.3 PDGF‐D Increased NOTCH1 Expression in Glioma Cells by Upregulating NF‐κB p‐p65

**FIGURE 4:**
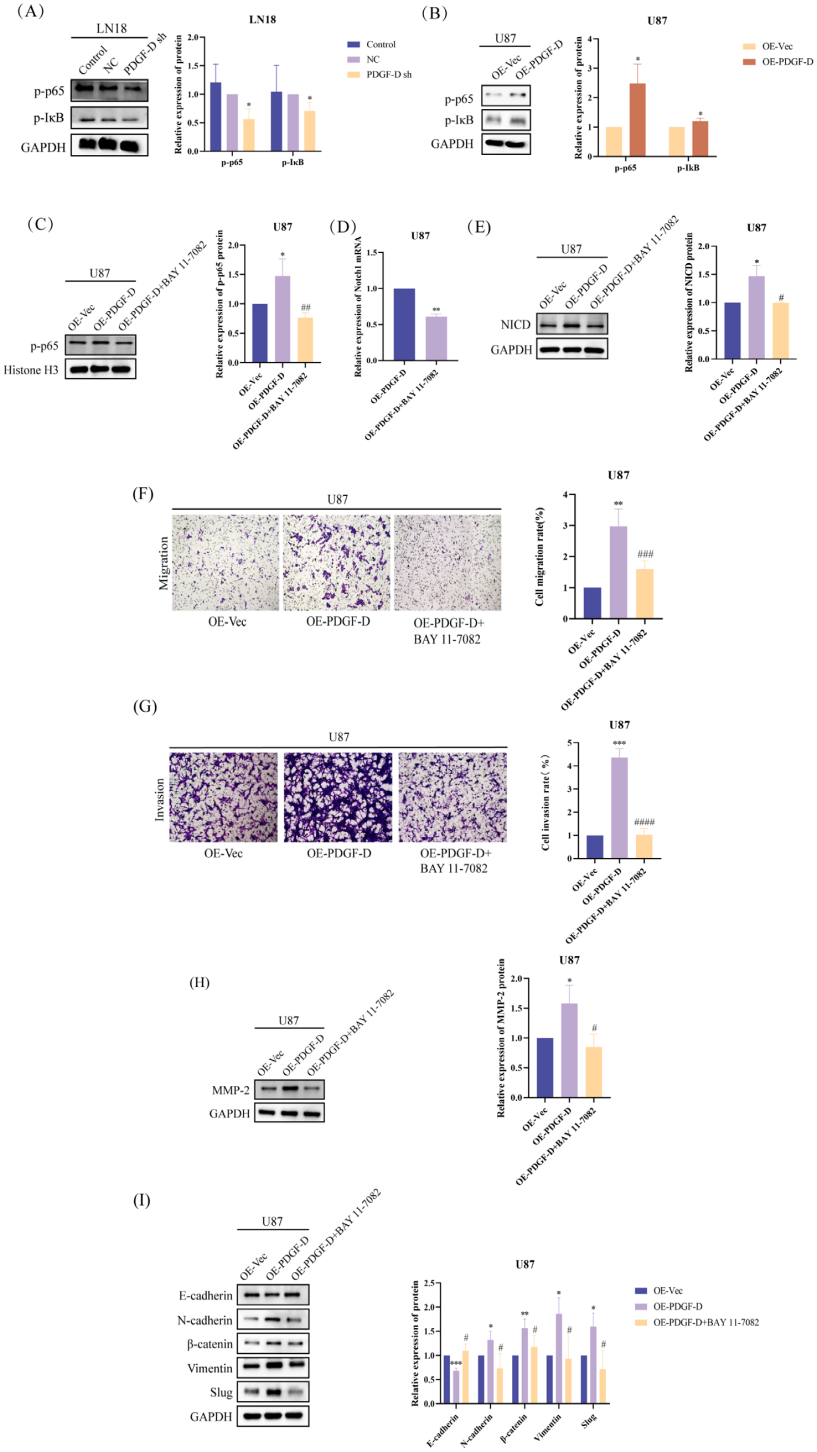
PDGF‐D upregulated NOTCH1 by upregulating the phosphorylation of p65. (A) Effect of PDGF‐D knockdown on the levels of p‐IκB and p‐p65 in LN18 cells. (B) Effect of PDGF‐D overexpression on the levels of p‐IκB and p‐p65 in U87 cells. (C) Effect of BAY 11–7082 on nuclear p‐p65 levels in U87 cells overexpressing PDGF‐D. (D, E) Effect of BAY 11–7082 on NOTCH1 mRNA and NICD protein expression in U87 cells overexpressing PDGF‐D. (F, G) Effect of BAY 11–7082 on the migration and invasion of U87 cells overexpressing PDGF‐D. (H, I) Effect of BAY 11–7082 on the expression levels of MMP‐2 and EMT‐related proteins in U87 cells overexpressing PDGF‐D. *p < 0.05, **p < 0.01, ***p < 0.001 vs. NC or OE‐Vec. ^#^ *p* < 0.05, ^##^ *p* < 0.01, ^###^ *p* < 0.001, ^####^ *p* < 0.0001 vs. OE‐PDGF‐D.

PDGF‐D knockdown significantly downregulated NF‐κB p‐p65 and p‐I‐κB in the LN18 cells (*p* < 0.05) (Figure 4A), while overexpression of PDGF‐D increased the phosphorylation of both proteins (*p* < 0.05) (Figure 4B), indicating that PDGF‐D regulates NOTCH1 signaling in glioma cells through NF‐κB p‐p65. To further explore this hypothesis, we inhibited NF‐κB p65 phosphorylation in the PDGF‐D^OE^ U87 cells using BAY 11–7082. As shown in Figure 4C, BAY 11–7082 significantly reduced the levels of NF‐κB p‐p65 (*p* < 0.01). In addition, NOTCH1 mRNA (*p* < 0.01) and NCID protein (*p* < 0.05) expression levels were also significantly inhibited (Figure 4D,E). In line with our findings so far, inhibiting NF‐κB p65 phosphorylation with BAY 11–7082 significantly reduced the migration (*p* < 0.001) and invasion (*p* < 0.0001) of PDGF‐D^OE^ U87 cells compared to the untreated cells in the transwell assay (Figure 4F,G). Finally, MMP‐2 (*p* < 0.05) (Figure 4H) and mesenchymal markers (*p* < 0.05) (slug, vimentin, β‐catenin, and N‐cadherin) were downregulated, and E‐cadherin was upregulated (*p* < 0.05) (Figure 4I) after inhibiting the phosphorylation of NF‐κB p65. Taken together, PDGF‐D increased NOTCH1 expression in glioma cells by upregulating NF‐κB p‐p65.

### 3.4 PDGF‐D Overexpression Enhanced Glioma Growth and EMT In Vivo

**FIGURE 5:**
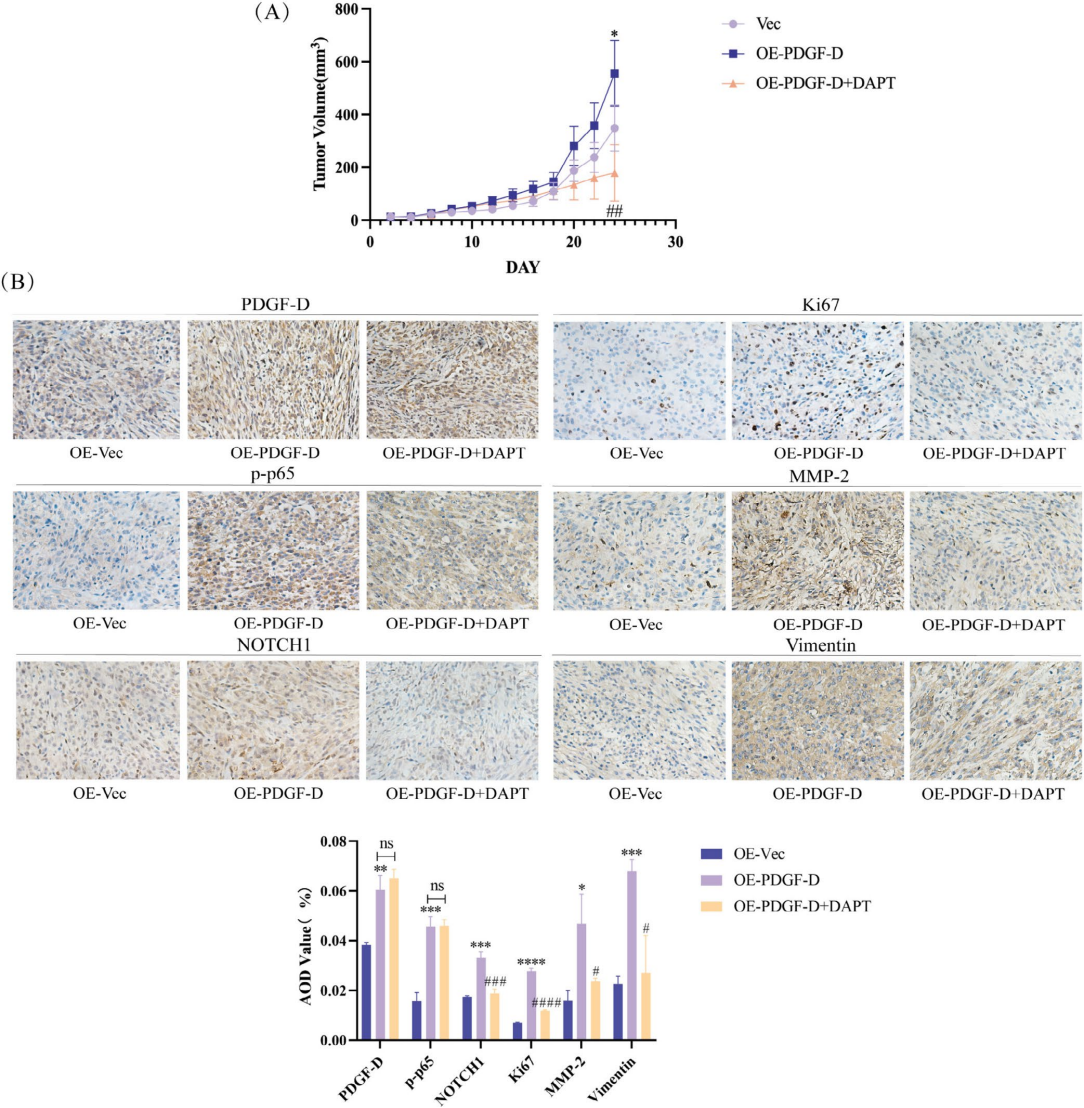
Results of the in vivo experiment. (A) The tumor‐bearing nude mice were injected intraperitoneally with 2.5 mg/mL DAPT (100 mg/kg) or equal volume of physiological saline starting on the 8th day after subcutaneous inoculation of tumor cells, and on alternate days thereafter. The volume of subcutaneous tumors was measured every other day (B) Representative immunostaining images of tumor tissues showing expression of the indicated proteins. The brown‐colored regions are positively stained. Images are 200× magnified. *p < 0.05, **p < 0.01, ***p < 0.001, ****p < 0.0001 vs. OE‐Vec. ^#^ *p* < 0.05, ^##^ *p* < 0.01, ^###^ *p* < 0.001, ^####^ *p* < 0.0001 vs. OE‐PDGF‐D.

To verify the in vitro findings, we established a subcutaneous model of glioma in mice using control and PDGF‐D^OE^ U87 cells. As shown in Figure 5A, the PDGF‐D^OE^ cells formed significantly larger tumors compared to the control cells (*p* < 0.05). However, intraperitoneal injection of DAPT into the PDGF‐D^OE^ glioma‐bearing mice markedly reduced the tumor volume relative to that in the untreated mice (*p* < 0.01) (Figure 5A). Furthermore, the in situ expressions of PDGF‐D (*p* < 0.01), NF‐κB p‐p65 (*p* < 0.001), NOTCH1 (*p* < 0.001), Ki67 (*p* < 0.0001), MMP‐2 (*p* < 0.05), and vimentin (*p* < 0.001) were also significantly higher in xenografts derived from the PDGF‐D^OE^ cells compared to that of the control group but decreased in the tumors of DAPT‐treated mice (*p* < 0.05). However, DAPT did not significantly impact the expression levels of PDGF‐D and NF‐κB p‐p65 (Figure 5B).

## 4 Discussion

PDGF‐B was long considered the key protein of the PDGF family involved in glioma progression, until [22] Lokker et al. reported that PDGF‐D is ubiquitously expressed in glioma tissues rather than PDGF‐B, suggesting its potential role in promoting glioma development. Consistent with previous reports, PDGF‐D was upregulated in the glioma tissues compared to normal brain tissues and correlated with the malignant phenotype and poor prognosis. Furthermore, PDGF‐D also induced EMT in glioma cells, which is critical for their migration and invasive behavior [11, 21, 33].

PDGF‐D has multiple downstream effectors, including p38, NOTCH1, Bcl‐2, miR‐106a, etc. [21, 34, 35, 36, 37]. Dll4, one of the ligands of NOTCH1, promotes its cleavage and activation [38]. Consistent with this, PDGF‐D knockdown or overexpression in glioma cells led to corresponding changes in the expression of NOTCH1 protein, and NOTCH1 activation using Dll4 reversed the suppressive effects of PDGF‐D knockdown on the migration and invasion of LN18 cells. Likewise, inhibition of NOTCH1 cleavage with DAPT, a γ‐secretase blocker, neutralized the pro‐oncogenic effects of PDGF‐D overexpression on the U87 cells. These findings suggested that NOTCH1 mediates the oncogenic effects of PDGF‐D in glioma.

PDGF upregulation also activates the NF‐κB pathway [39]. Yu et al. showed that knocking down PDGF‐D in esophageal squamous cell carcinoma cells downregulated the p‐IκB and p65 proteins, and the resulting inactivation of the NF‐κB pathway inhibited cell proliferation and invasion [35]. Furthermore, Zhou et al. demonstrated that NF‐κB directly interacts with the promoter region of NOTCH1 through dual luciferase reporter experiments [31]. Therefore, we hypothesized that PDGF‐D promotes the EMT of glioma cells by activating the NOTCH1 signaling pathway, which is dependent on NF‐κB p65 phosphorylation and nuclear translocation. In this study, we inhibited NF‐κB p65 phosphorylation in U87 cells overexpressing PDGF‐D using BAY 11–7082 and detected a significant reduction in NCID levels. In addition, NF‐κB inactivation significantly inhibited the EMT, migration, and invasion of U87 cells. This hypothesis concurs with previous studies showing that PDGF‐D upregulates NOTCH1 and activates downstream signaling pathways, thereby indirectly amplifying EMT [40, 41, 42].

We validated the in vitro findings by establishing subcutaneous tumors in BALB/c‐nu mice [43]. The tumors overexpressing PDGF‐D were markedly larger than the control tumors, and DAPT treatment inhibited the growth of the PDGF‐D^OV^ tumors. The decrease in proliferation was confirmed by the downregulation of Ki67 in the tumor tissues. Ectopic expression of PDGF‐D also increased the levels of NF‐κB p‐p65, NOTCH1, and mesenchymal markers in the tumor tissues, and these changes were reversed by DAPT treatment. Taken together, the PDGF‐D/NF‐κB/NOTCH1 axis regulates the migration, invasion, and EMT of glioma cells.

## Author Contributions

Y.L.: Conceptualization; investigation; writing – original draft. Y.Z.: Conceptualization; methodology, writing – review and editing. M.S. : Resources; software; visualization. X.M.: Data curation; software. M.J.: Data curation; formal analysis. Z.S.: Investigation; writing – original draft. X.L.: Formal analysis; investigation. Y.L.: Funding acquisition; supervision; validation. L.Z.: Funding acquisition; project administration; supervision.

## Disclosure

Clinical Perspectives: PDGF‐D regulates the migration, invasion, and EMT of a variety of tumor cells, but its role in glioma, a malignancy with a very poor prognosis, is not completely clear. Our results confirm that PDGF‐D is highly expressed in glioma tissues and correlates with the malignant phenotype and poor prognosis. It promotes EMT, migration, and invasive behavior of glioma cells by activating the NF‐κB/NOTCH1 pathway, which can potentially offer therapeutic targets for the development of new drugs.

Animal Studies: The study was approved by the Ethics Committees of The First Hospital of Jilin University (20230637–29/08/2023).

## Ethics Statement

Approval of the research protocol by an Institutional Review Board: The study was approved by the Ethics Committees of The Second Hospital of Jilin University and The First Hospital of Jilin University (2019080–20/09/2019 and 19 K127‐10/09/2020, respectively).

## Conflicts of Interest

The authors declare no conflicts of interest.

## Data Availability

The datasets used and/or analyzed during the current study are available from the corresponding author on reasonable request.
